# Afadin requirement for cytokine expressions in keratinocytes during chemically induced inflammation in mice

**DOI:** 10.1111/gtc.12184

**Published:** 2014-10-09

**Authors:** Toshiyuki Yoshida, Takanori Iwata, Yoshimi Takai, Walter Birchmeier, Masayuki Yamato, Teruo Okano

**Affiliations:** 1 Institute of Advanced Biomedical Engineering and Science, Tokyo Women's Medical University8-1 Kawada-cho Shinjuku-ku, Tokyo, 162-8666, Japan; 2 Division of Pathogenetic Signaling, Department of Biochemistry and Molecular Biology, Kobe University Graduate School of Medicine1-5-6 Minatojima-minamimachi, Chuo-ku Kobe, Hyogo, 650-0047, Japan; 3 Max Delbrück Center for Molecular MedicineRobert-Rössle-Str. 10, Berlin-Buch, 13125, Germany

## Abstract

Afadin is a filamentous actin-binding protein and a mediator of nectin signaling. Nectins are Ig-like cell adhesion molecules, and the nectin family is composed of four members, nectin-1 to nectin-4. Nectins show homophilic and heterophilic interactions with other nectins or proteins on adjacent cells. Nectin signaling induces formation of cell–cell junctions and is required for the development of epithelial tissues, including skin. This study investigated the role of afadin in epithelial tissue development and established epithelium-specific afadin-deficient (CKO) mice. Although showing no obvious abnormality in the skin development and homeostasis, the mice showed the reduced neutrophil infiltration into the epidermis during chemical-induced inflammation with 12-*O*-tetradecanoylphorbol 13-acetate (TPA). Immunohistochemical and quantitative real-time PCR analyses showed that the expression levels of cytokines including Cxcl2*,* Il-1β and Tnf-α were reduced in CKO keratinocytes compared with control keratinocytes during TPA-induced inflammation. Primary-cultured skin keratinocytes from CKO mice also showed reduced expression of these cytokines and weak activation of Rap1 compared with those from control mice after the TPA treatment. These results suggested a remarkable function of afadin, which was able to enhance cytokine expression through Rap1 activation in keratinocytes during inflammation.

## Introduction

Mammalian skin is composed of an epidermis, dermis, and its appendages including hair follicles and sweat glands. The epidermis is divided into four layers. From the top (outer-most side), these layers are the cornified, granular, papillary and basal layers (Dai & Segre 2004). Skin mainly acts as a physical barrier to the outer environment, and this function is mainly owing to the highly keratinized cornified layer and tight junctions between the cornified and granular layers. In addition to the barrier function, previous studies have showed various functions of the epidermis, such as immunity. Immune cells including αβ T-cells, γδ T-cells and Langerhans cells are in the epidermis and play roles in antigen presentation and cytokine expression (Heath & Carbone 2013). Keratinocytes, the main cell component of the epidermis, also have an immunological function. Keratinocytes detect pathogens through Toll-like, Nod-like, RIG-I-like receptors, and secrete antimicrobial peptides such as LL-37, β-defensin family proteins, RNases and S100 proteins (Heath & Carbone 2013). Additionally, keratinocytes secrete cytokines against bacterial and chemical stimuli. Both *in vivo* and *in vitro*, keratinocytes secrete various cytokines including pro-inflammatory, T-cell-trophic and immunomodulatory cytokines, and are found to recruit inflammatory cells including neutrophils and T cells (Grone 2002).

Afadin is a filamentous actin-binding protein that accumulates at the cell–cell junctions in the polarized epithelium and is composed of functional domains interacting with other proteins (Takai *et al*. 2008). Among these afadin-interacting proteins, interactions with nectin family proteins have been intensively studied. The nectin family proteins are immunoglobulin-like cell adhesion molecules; the family is composed of four members, nectin-1, nectin-2, nectin-3 and nectin-4 (Takai *et al*. 2008). Nectins show hemophilic and heterophilic interactions with other nectins or other proteins on adjacent cells, and the nectin interaction induces the formation of adherens junctions, and then tight junctions. Afadin interacts with all four nectins via their PDZ domain and connects nectins to the actin cytoskeleton. Afadin is required for nectin signaling through interactions with α-catenin at adherens junctions and ZO-1 at tight junctions (Takai *et al*. 2008).

Mutations in the human *NECTIN-1/PVRL1* gene have been found to be responsible for human ectodermal dysplasia (ED), which is characterized by cleft lip/palate, syndactyly, abnormal hair and missing teeth (Zlotogora *et al*. 1987; Ogur & Yuksel 1988; Zlotogora 1994; Suzuki *et al*. 2000; Sozen *et al*. 2001; Brancati *et al*. 2010). Because junctional formation plays an important role in epithelial tissue development, afadin- and several nectin-deficient mice have been established. Mouse strains deficient in single nectins are viable and show abnormal formations of ectodermal tissues including eye, tooth, inner ear and skin (Inagaki *et al*. 2005; Wakamatsu *et al*. 2007; Barron *et al*. 2008; Brancati *et al*. 2010; Togashi *et al*. 2011). In all tissues, the aberrant formation of adherens, tight, and desmosomal junctions are observed, and are thought to be the cause of these abnormalities. A compound nectin-1 and nectin-3 mutant mouse shows malformed teeth and dies approximately postnatal day 10 (Yoshida *et al*. 2010, 2014). However, none of the nectin mutant strains shows missing teeth, cleft lip/plate or syndactyly, which are ectodermal abnormalities observed in human ED patients.

Mice lacking afadin show abnormal formation of adherens junctions and die approximately embryonic day 7.5 (Ikeda *et al*. 1999). To investigate the role of afadin in the later stages, several afadin conditional knockout mice lines have been generated. *Nestin-Cre-*mediated central nervous system-specific afadin-deficient mice exhibit hydrocephalus and die by postnatal day 21 (Yamamoto *et al*. 2013). *Emx1-Cre*-mediated central nervous system-specific afadin-deficient mice develop a double cortex, a brain malformation in which heterotopic gray matter is interposed between zones of white matter (Gil-Sanz *et al*. 2014) (H. Yamamoto, K. Mandai, and Y. Takai, unpublished data). *CamK-Cre-*imediated hippocampal neuron-specific afadin-deficient mice are viable and show disruption of puncta-adherens junctions (Majima *et al*. 2009). *Villin-Cre-*mediated intestinal epithelia-specific afadin-deficient mice show increased permeability in the intestinal mucosa and enhanced susceptibility to tissue destruction induced by dextran sulfate sodium (Tanaka-Okamoto *et al*. 2011). In all conditional afadin-deficient mouse lines, disrupted cell–cell junctions, including adherens and tight junctions, are observed.

This study generated the epithelium-specific afadin conditional knockout (CKO) mice. The aim of this study was to investigate the function of afadin in skin physiology and pathogenesis.

## Results

**Figure 1:**
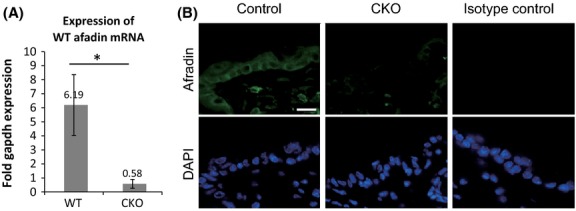
Expression of afadin in control and afadin conditional knockout (CKO) skin. (A) Expression of wild-type afadin mRNA in adult mouse tail skin. Afadin mRNA expression in 8-week-old mouse tail skins is analyzed by SYBR Green-based real-time RT-PCR. The primers for afadin are designed against the 2nd exon, which is eliminated in the mutant allele. The bars and lines represent the means and standard deviations of three skin samples (*P < 0.01). (B) Immunohistochemistry of afadin in adult back skin. Nuclei are stained with DAPI. Scale bar: 25 μm.

**Figure 2:**
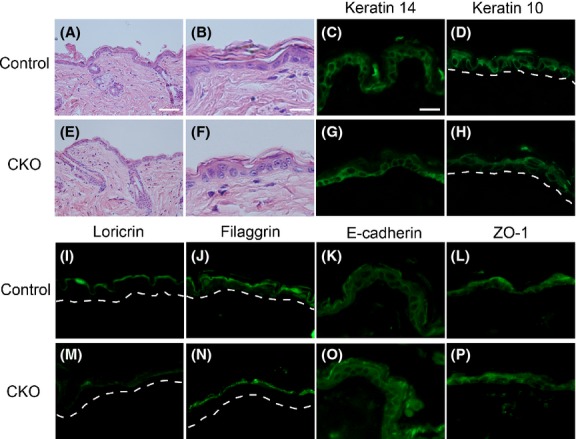
Histological analysis of control and afadin conditional knockout (CKO) skin. (A, B, E, F) Hematoxylin and eosin staining of adult control (A, B) and afadin CKO (E, F) skin. A and E are lower, and B and F are higher magnification photographs. (C, D, G–P) Immunohistochemistry of adult control (C, D, I–L) and CKO (G, H, M–P) skin using the antibodies shown at the top of each photograph. Dotted lines in G and J indicate basement membrane. Scale bars: 80 μm in A, E, 25 μm in B, C, D, F–P.

This study crossed keratin 14 (K14)-derived *Cre*-expressing mice, which expressed *Cre* in the epithelium from embryonic day 15, with afadin floxed mice, and created K14 *Cre* afadin flox/flox (CKO) mice. In these mice, the expression of normal afadin mRNA in tail skin and expression of afadin protein in back skin were severely reduced (Fig.1A, B). The CKO mice were viable, grew normally, and were fertile. The development of epithelial tissues, including skin and salivary glands, occurred normally (Fig.2A, B, E, F). The teeth of CKO mice showed mild hypomorpy (Fig. S1 in Supporting Information).

The expression of skin differentiation markers including K14, K10 and filaggrin, which are expressed in the basal, papillary and cornified layers, respectively, in CKO skin, was comparable to that in K14 *Cre Afadin* flox/+ (control) mice (Fig.2C, D, G, H, J, N). The expression of loricrin, a marker of the granular layer, was slightly reduced in CKO mouse skin compared with that in control mice (Fig.2I, M). The expression and localization of junctional proteins including E-cadherin and ZO-1, components of adherens and tight junctions, respectively, in CKO skin, were also comparable to those in control mice (Fig.2K, L O, P).

**Figure 3:**
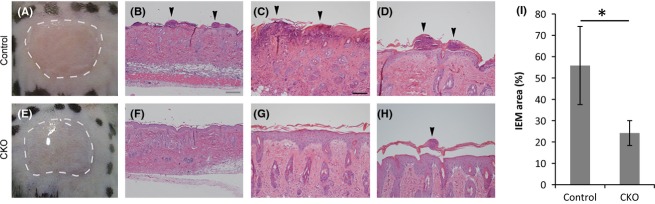
Mouse back skin treated with TPA. (A, E) Macroscopic images of TPA-treated skin. Images captured 5 min after the first application of TPA onto denuded back skin of adult control (A) and CKO (E) mice. Dotted lines indicate TPA solution. (B, C, D, F, G, H) Hematoxylin and eosin stained skin specimens of skin treated with 12-*O*-tetradecanoylphorbol 13-acetate (TPA) for 3 days. Lower (B, F) and higher (C, D, G, H) magnification photographs of the control (A–D) and afadin conditional knockout (CKO) (E–H) back skin. Arrowheads indicate cells that infiltrated the epidermis. (I) Average IEM regions in TPA-treated skin. The bars and lines represent the means and standard deviations of seven mouse skin samples (*P < 0.01). Scale bars: 100 μm in B, F, 50 μm in C, D, G, H.

**Figure 4:**
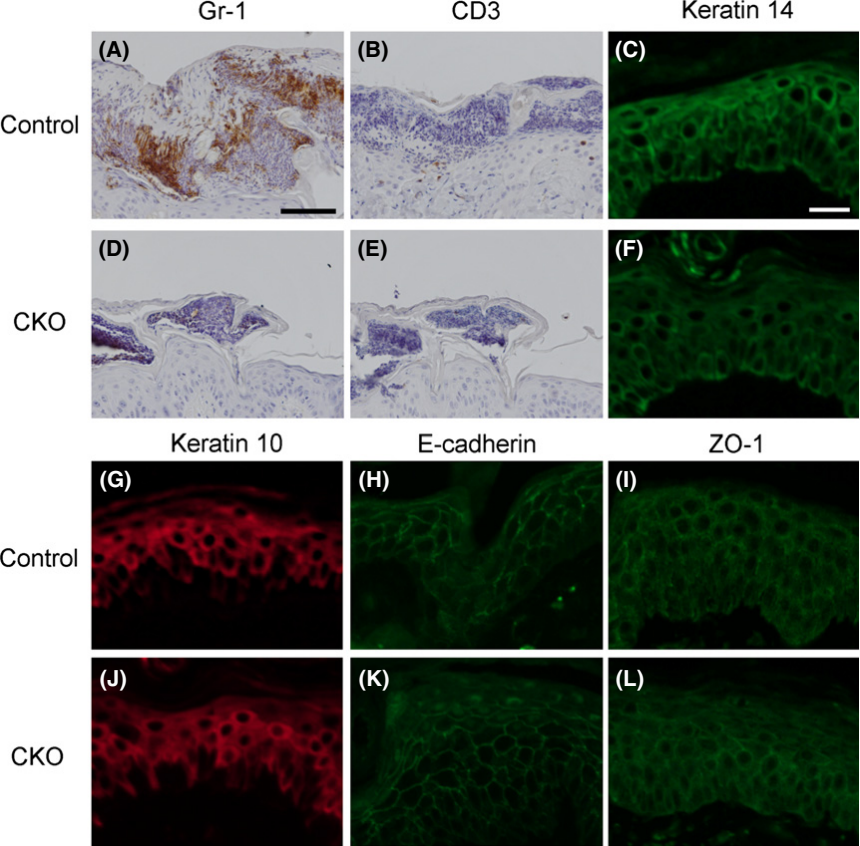
Immunohistochemical analysis of 12-*O*-tetradecanoylphorbol 13-acetate (TPA)-treated skin specimens. TPA-treated skins from control (A–C, G–I) and afadin conditional knockout (CKO) (D–F, J–L) mice were stained with the antibodies shown at the top of each photograph. Scale bars: 50 μm in A, B, D, E, 25 μm in C, F–L.

The adult mouse back was denuded, and 12-*O*-tetradecanoylphorbol 13-acetate (TPA) was applied onto the back skin once a day for 3 days (Fig.3A, E). The TPA treatment induced skin inflammation and the infiltration of inflammatory cells into the epidermis (intra-epidermal microabscess, IEM) in the skin (Fig.3B, C, D). In CKO mice, the cell infiltrated area was smaller than that of control skin after TPA treatment (Fig.3F–I). Immunohistochemistry showed that the cells in IEMs were mostly neutrophils (Gr-1 positive) and that there were few T cells (CD3 positive) (Fig.4A, B). IEMs in CKO skin showed severely reduced neutrophil infiltration (Fig.4D, E). Although an obvious abnormality in the formation of IEMs was observed in CKO skin, the expression and localization of skin differentiation markers and junctional proteins were comparable to those in control skin (Fig.4C, F–L).

**Figure 5:**
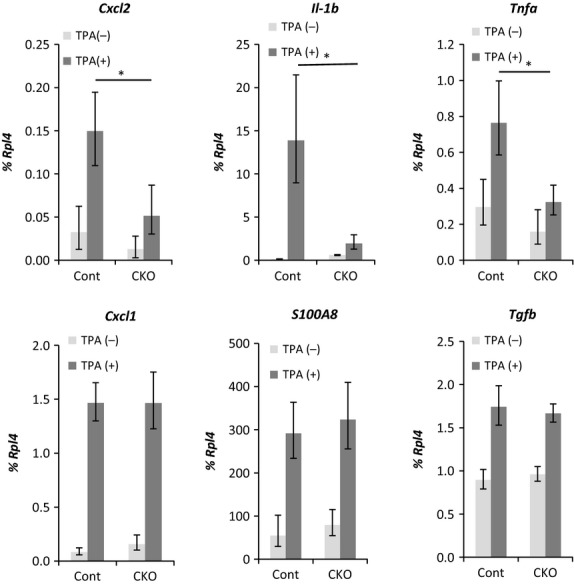
Gene expressions in 12-*O*-tetradecanoylphorbol 13-acetate (TPA)-treated skin specimens from control and afadin conditional knockout (CKO). Expression levels of the indicated genes shown at the top of each graph in skin treated with TPA for 3 days were analyzed by real-time RT-PCR. The bars and lines represent the means and standard deviations of seven mouse skin samples (*P < 0.01).

**Figure 6:**
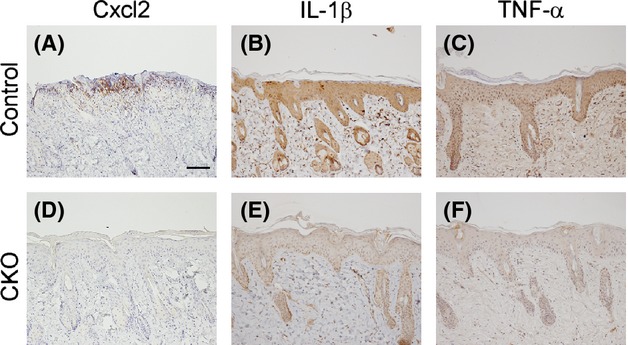
Immunohistochemical analysis of 12-*O*-tetradecanoylphorbol 13-acetate (TPA)-treated skin. TPA-treated skin from control (A–C) and afadin conditional knockout (CKO) (D–F) mice was stained with the antibodies shown at the top of each picture. Scale bar 50 μm.

**Figure 7:**
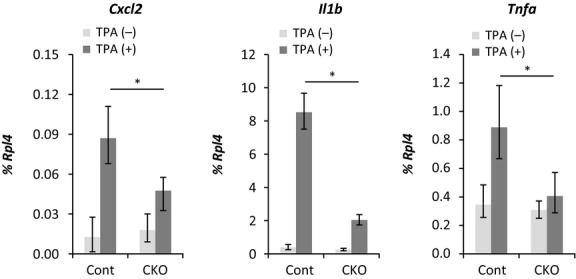
Gene expressions levels in 12-*O*-tetradecanoylphorbol 13-acetate (TPA)-treated skin specimens from control and afadin conditional knockout (CKO) mice. The expression levels of the indicated genes shown at the top of each graph in skin treated once with TPA were analyzed by real-time RT-PCR. The bars and lines represent the means and standard deviations of seven mouse skin samples (*P < 0.01).

RNA was extracted from skin treated with TPA for 3 days, and the expression levels of inflammatory cytokines including *Cxcl1*,*Cxcl2*,*IL-1*β, *S100a8*,*Tgf-*β and *Tnf-*α were investigated using real-time RT-PCR. Among these genes, the expression levels of *Cxcl2*,*IL-1*β and *Tnf-*α were reduced in CKO skin compared with the levels in the control skin (Fig.5). Immunohistochemical analysis using skin treated with TPA for 3 days confirmed that Cxcl2, Il-1β and Tnf-α were expressed in keratinocytes and that the expression levels were reduced in CKO skin compared with control skin (Fig.6). The induction of *Cxcl2, Il-1*β *and Tnf-*α was already observed 2 h after the first application of TPA, and expression of these transcripts was reduced in CKO skin compared with control skin (Fig.7).

**Figure 8:**
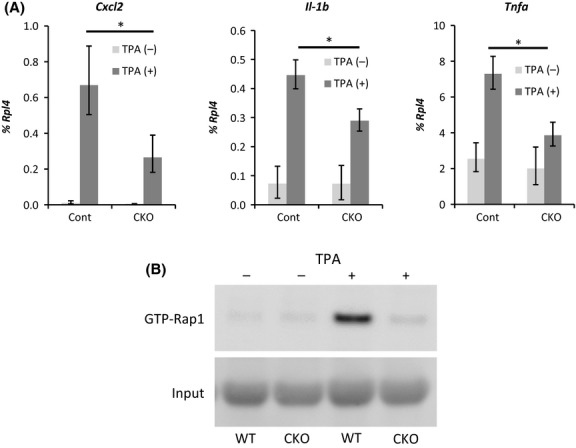
Effect of 12-*O*-tetradecanoylphorbol 13-acetate (TPA) treatment on cultured keratinocytes. (A) The expression levels of the genes shown at the top of each graph in keratinocytes treated with TPA for 2 h were analyzed by real-time RT-PCR. The bars and lines represent the means and standard deviations in primary keratinocytes from seven mice (*P < 0.01). (B) GTP-Rap1 pull-down assay from keratinocyte protein extracts with or without TPA treatment. The picture is the representative of three independent experiments.

Then, the effect of afadin deficiency on cytokine expression was investigated in keratinocytes *in vitro*. Keratinocytes were harvested from neonatal mouse back skin and treated with TPA. The expression of *Cxcl2*,*IL-1*β and *Tnf-*α was induced by TPA treatment, and the expression levels of these genes were lower in CKO keratinocytes than in the control keratinocytes (Fig.8A). Although the amount of the GTP-bound active form of Rap1, an afadin-interacting protein, was increased by TPA treatment, only a slightly increase was found in CKO keratinocytes (Fig.8B).

## Discussion

The CKO mice showed no abnormality in epithelial tissue formation and homeostasis. Because afadin-deficient mice show a failure of adherens junction formation and the deficiency was early embryonic lethal (Ikeda *et al*. 1999), several tissue-specific afadin deletion mouse lines were created to investigate the tissue-specific function of afadin. All mouse lines show abnormal tissue formation caused by the disrupted cell–cell junctions. Because K14 is expressed in the epithelium of skin, mouth, esophagus, trachea and upper stomach, the CKO mice were thought to show developmental defects in these tissues. However, the CKO mice showed no obvious skin abnormality in physiological condition. The CKO mice showed mild tooth abnormalities, but no obvious abnormality of the salivary gland. The epidermis and its appendages were formed and renewed normally, and differentiation markers and junctional proteins were expressed normally in CKO skin. This discrepancy suggested two possibilities. One was a compensation mechanism with other intracellular binding partners acting in the epidermis. Previous studies have shown that nectins interact with various proteins through their cytoplasmic domains (Takai *et al*. 2008). Nectin-interacting protein(s) other than afadin may transduce nectin signaling in afadin-deficient cells *in vivo*. The other possibility was that afadin was necessary for establishing junctions or initiating epithelial tissue formation, but unnecessary for later developmental and maintenance processes. In the *K14 Cre* mice, *Cre* was expressed from E15.5 (Huelsken *et al*. 2001) after initiation of formation of these tissues. Earlier depletion of afadin in these tissues might result in developmental defects.

As a previous study has shown that colon-specific afadin-deficient mice show abnormal inflammatory responses (Tanaka-Okamoto *et al*. 2011), this study applied an inflammatory chemical to the skin. The CKO mice showed reduced formation of IEMs in the skin after TPA treatment. TPA is a phorbol ester that acts as an analogue of diacylglycerol and elicits protein kinase C (PKC) signaling, and the activation of PKC signaling induces inflammation (Takai *et al*. 1984; Dotto 1999). Skin inflammation causes thickening of the epidermis, dedifferentiation of epidermal cells, junctional rearrangement, cytokine expression and formation of IEMs (Wang & Smart 1999). Although skin thickening and dedifferentiation and junctional reorganization of keratinocytes in the CKO skin were comparable to those seen in control skin, the formation of IEMs was disturbed in CKO skin. Immunohistochemistry results suggested the cells in IEMs were mostly neutrophils, consistent with the previous results showing that IEMs contain neutrophils (Wang & Smart 1999; Sano *et al*. 2005). Invasion of neutrophils into the epidermis is thought to be required for the interaction of cell-surface molecules between keratinocytes and neutrophils, similar to the interaction between endothelial cells and neutrophils (Wang & Cheng 2007). Because afadin is involved in the formation of tight junctions (Takai *et al*. 2008) and some tight junction proteins are involved in the invasion of neutrophils (Chin & Parkos 2007), the abnormal expression or localization of tight junction proteins in the CKO epidermis was a good candidate cause of the impaired invasion of neutrophils. However, immunohistochemistry results showed that the expression and localization of tight junction proteins in TPA-treated CKO skin were comparable to those in control skin, suggesting that loss of afadin unaffected junctional reorganization under an inflammatory situation and with invasion of neutrophils.

Cytokine expression levels in TPA-treated skin were lower in afadin-deficient keratinocytes than in control keratinocytes. Quantitative RT-PCR analysis of RNA extracted from skin treated with TPA showed that the expression levels of *Cxcl1*,*Cxcl2*,*IL-1*β, *S100a8*,*Tgf-*β and *Tnf-*α were up-regulated under conditions of TPA treatment and that those of *Il-1*β, *Cxcl2* and *Tnf-*α were reduced in CKO skin. The skin samples used in this study contained not only keratinocytes, but also other cell types, including dermal fibroblasts, Langerhans cells and tissue-residing T-cells, which also secrete cytokines (Heath & Carbone 2013). Therefore, this study carried out immunohistochemical analyses of these cytokines and RT-PCR using primary-cultured keratinocytes treated with TPA. The results suggested keratinocytes expressed these cytokines and that the expressions levels were reduced in CKO skin. The results of skin and keratinocyte cytokine expression analyses 2 h after the first treatment with TPA indicated that this cytokine induction was a direct effect of TPA and that the induction required afadin. Previous studies have showed that keratinocytes express cytokines during inflammation and induced migration of inflammatory cells including neutrophils (Cybulsky *et al*. 1988; Grone 2002; Christensen & Haase 2012). The cytokines reduced in the CKO mice are known to be pro-inflammatory cytokines inducing neutrophil migration (Cybulsky *et al*. 1988; Grone 2002; Christensen & Haase 2012). Therefore, afadin was speculated to be required for the expression of inflammatory cytokines including Cxcl2, Il-1β and Tnf-α in keratinocytes, and the reduced migration found in neutrophils in CKO mice was likely to be caused by the reduced expression of these cytokines.

Recent studies have reported the immunological function of nectin signaling (Chan *et al*. 2012; Chen & Flies 2013). Nectins and nectin-likes (Necls) are expressed in tumors and antigen-presenting cells and their ligands are expressed on cytotoxic T cells and NK cells. CD226 interacts with nectin-2 and Necl-5. T-cell immunoreceptor with immunoglobulin and ITIM domains (TIGHT) interacts with Necl-5, nectin-2 and nectin-3. Cytotoxic and regulatory T-cell molecule (CRTAM) interacts with Necl-2. CD96 interact with nectin-1. These interactions act as cosignaling pathways and modulate the activation after antigen recognition and cytotoxic activity of T cells and NK cells. The functions of these interactions are to mediate direct cell–cell interactions and affect ligand-expressing T cells and NK cells. However, the function of afadin as suggested by the results of this study was indirect. These results suggested the possibility of additional afadin functions in immunological processes.

Previous studies have shown that afadin interacts with Rap1 (Hoshino *et al*. 2005; Takai *et al*. 2008), which is involved in the expression of cytokines in keratinocytes (Grader-Beck *et al*. 2003; Ikuta *et al*. 2008). Rap1 is a small G protein that belongs to the Ras GTPase superfamily. Rap1 has been shown to be involved in the establishment of cell adhesion (Hoshino *et al*. 2005; Choi *et al*. 2013). Rap1 binds either GDP or GTP, and the GDP-bound form (GDP-Rap1) and GTP-bound form (GTP-Rap1) are the inactive and the active form, respectively. (Kooistra *et al*. 2007). A pull-down assay of GTP-Rap1 using protein extracted from primary-cultured keratinocytes showed that TPA treatment induced activation of Rap1 and that the activation was severely reduced in CKO keratinocytes. Weak activation of Rap1 has also been observed in afadin-deficient intestinal epithelium (Tanaka-Okamoto *et al*. 2011). There were two possible mechanisms by which the lack of afadin caused the loss of GTP-Rap1. One was that afadin directly interacted with Rap1 and that this interaction was required for the conversion of Rap1 from the GDP-bound inactive form to the GTP-bound active form. The other was that afadin suppressed the activity of GTPase activating protein (GAP), which converted GTP-Rap1 to GDP-Rap1. A previous study has showed that afadin interacts with signal-induced proliferation-associated protein-1 (SPA-1), a Rap1-specific GAP, and modulates Rap1 activity (Su *et al*. 2003). Furthermore investigation is required to evaluate these two possible mechanisms. Taken together, these results suggested (1) the cytokine expression in TPA-treated keratinocytes required afadin and that (2) the stimulation of the activation of Rap1 and/or the inhibition of the inactivation of Rap1 required afadin.

This study suggested afadin was involved in cytokine expression during the first step of inflammation. Previous studies have reported that afadin plays roles in a variety of cell functions, such as junctional formation, cytoskeletal reorganization, cell polarization, cell migration, cell differentiation, cell survival and immunological responses (Takai *et al*. 2008). Our results proposed a novel function of afadin, which was required for the expression of cytokine during inflammation. Although the activation of Rap1 was reduced in the afadin-deficient situation, the mechanism by which afadin signaling induced the cytokine expression remained largely unknown. Furthermore investigations are required to improve our understanding of this phenomenon.

## Experimental procedures

### Ethics statement

All of the animal experiments were carried out according to the ‘Guidelines of Tokyo Women's Medical University on Animal Use’, the ‘Principles of Laboratory Animal Care’ formulated by the National Society for Medical Research, and the ‘Guide for the Care and Use of Laboratory Animals’ prepared by the Institute of Laboratory Animal Resources and published by the National Institutes of Health (NIH Publication No. 86-23, revised 1985). All of the animals were treated according to the experimental procedures approved by the Committee for Animal Research of Tokyo Women's Medical University in Tokyo, Japan (Permit Number: 11-125).

### Animals

Keratin 14 (K14) Cre mice were provided by Prof. Birchmeier (Huelsken *et al*. 2001) and the afadin flox/flox mice were generated as previously described (Majima *et al*. 2009). The mice were kept and crossed at K.A.C. Corporation (Tokyo, Japan). Mice were kept in specific pathogen free condition, with 12 h light/12 h dark cycle and temperature at 21 °C. The mice were euthanized using CO_2_ followed by cervical dislocation (adult) or decapitation (newborn).

### Tissue preparation, histology and immunohistochemistry

Mouse tissues were fixed with 4% paraformaldehyde before embedding in paraffin. Sections were sliced at 5 μm, and immunostaining was carried out as previously described (Yoshida *et al*. 2010). The following primary antibodies were used at the indicated dilutions after performing antigen retrieval using antigen unmasking solution for 10 min (H3300) (Vector Laboratories, Burlingame, CA, USA): CD3 at 1:100 (M3070) (Spring Bioscience, Pleasanton, CA, USA), Cxcl2 at 1:100 (AAM48) (AbD SEROTEC, Oxford, U.K.), E-cadherin at 1:200 (13900) (Life Technologies, Carlsbad, CA, USA), filaggrin at 1:200 (PRB-417P) (Covance, Princeton, NJ, USA), Gr-1 at 1:200 (108403) (BioLegend, San Diego, CA, USA), keratin 10 at 1:400 (PRB-159P) (Covance), keratin 14 at 1:250 (RB-9020-P0) (Thermo Scientific, Waltham, MA, USA), Il-1β at 1:100 (ab9722) (abcam, Cambridge, UK), Tnf-α at 1:100 (250844) (Abbiotech, Gatineau, Canada), ZO-1 at 1:100 (61-7300) (Life Technologies) and L/s-afadin at 1:100 (A 0224) (Sigma, Saint Louis, MO, USA).

### Treatment with 12-*O*-tetradecanoylphorbol 13-acetate

Application of 12-*O*-tetradecanoylphorbol 13-acetate (TPA) (BML-PE160) (Enzo Life Sciences, Farmingdale, NY, USA) onto 6- to 10-month-old mouse back skin was carried out as previously described with some modifications (Kaya *et al*. 2006). The mice were kept under inhalation anesthesia using isoflurane during the following procedures. The back hair was removed using depilatory cream (epilat) (Kracie, Tokyo, Japan) 2 days before TPA application. TPA was dissolved in DMSO at 500 μg/mL and diluted with acetone at 50 μg/mL before application. A total of 50 μL of TPA solution was applied once, or once a day for 3 days. At 2 h after the final application of TPA, mice were euthanized, and the skin samples were collected. The average length of the IEM region was calculated from the IEM lengths in the selected 2-mm skin length at the center of TPA-treated skin.

### RNA extraction, cDNA synthesis and polymerase chain reaction (PCR)

RNA extraction, cDNA synthesis and PCR from the skin samples and cultured keratinocytes were carried out as previously described (Yoshida *et al*. 2012). To detect wild-type afadin mRNA, the following primers were used: *afadin* (forward, gaaggaagatcatagttgca; reverse, gtcatttctgtgcctgtaac) and *Gapdh* (forward, accacagtccatgccatcac; reverse, tccaccaccctgttgctgta). The following TaqMan probes were used: *Cxcl1* (Mm04207460_m1), *Cxcl2* (Mm00436450_m1), *Il-1*β (*Il1b*, Mm00434228_m1), *S100a8* (Mm00496696_g1), *Tgf-*β (Tgfb1, Mm00441724_m1), *Tnf-*α (*Tnf*, Mm00443260_g1) and *Rpl4* (Mm01171353_g1). *Rpl4* was used as an internal control gene.

### Keratinocyte culture

Collection of mouse keratinocytes from neonatal pups and primary culture of keratinocytes were carried out as previously described (Wakamatsu *et al*. 2007). TPA was applied onto passage 1 keratinocytes at 5 ng/mL and the cells were collected 2 h after TPA treatment for TaqMan assays and the GTP-Rap1 pull-down assay, which was carried out using an Active Rap1 Pull-Down and Detection Kit (16120) (Thermo Scientific) according to the manufacturer's instructions.

### Data analysis

All values are expressed as mean ± SD. All samples were analyzed using Student's *t*-tests, and probability values less than 0.01 (*P* < 0.01) were considered statistically significant.

## Supporting Information

**Figure S1** Tooth phenotype of afadin conditional deficient mice.
