# Supplementary figures and images for: Afadin requirement for cytokine expressions in keratinocytes during chemically induced inflammation in mice
Source: Genes Cells. 2014 Oct 9;19(11):842–52. doi: 10.1111/gtc.12184 (PMC4231224; doi:10.1111/gtc.12184)

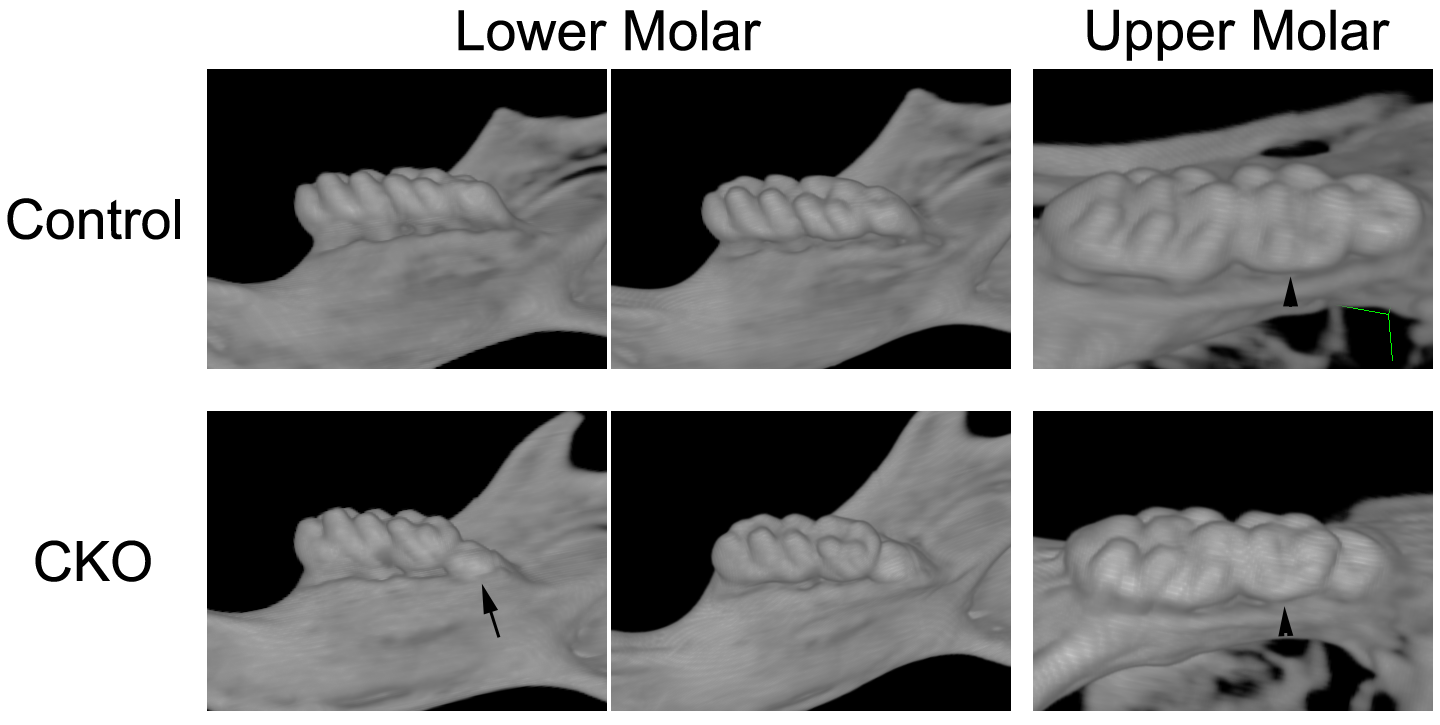

Supplement: Supplementary file 1 — Figure S1 Tooth phenotype of afadin conditional deficient mice. [file gtc0019-0842-sd1.tif]
