# Supplementary material for: Afadin requirement for cytokine expressions in keratinocytes during chemically induced inflammation in mice
Source: Genes Cells. 2014 Oct 9;19(11):842–52. doi: 10.1111/gtc.12184 (PMC4231224; doi:10.1111/gtc.12184)
Supplement: Supplementary file 2 [file gtc0019-0842-sd2.docx]

Figure S1

Tooth phenotype of afadin conditional deficient mice.

Scatter images of micro computed tomography of 2-week old control and afadin conditional deficient (CKO) mice. Third molar of lower jaw (arrow) is smaller in CKO mouse than that in control mouse. The cusp pattern of upper 2nd (arrowhead) is disrupted in CKO mouse compared with that in control mouse.
